# Disadvantages of Automated Respiratory Gas Exchange Analyzers

**DOI:** 10.3389/fphys.2020.00019

**Published:** 2020-02-07

**Authors:** Juan José Ramos-Álvarez, Irma Lorenzo-Capellá, Francisco Javier Calderón-Montero

**Affiliations:** ^1^School of Sports Medicine, Madrid Complutense University, Madrid, Spain; ^2^Faculty of Education and Health, Camilo José Cela University, Madrid, Spain; ^3^Faculty of Physical Activity and Sport Sciences, INEF, Madrid Polytechnic University, Madrid, Spain

**Keywords:** ergospirometry, oxygen uptake, carbon dioxide output, gas exchange analyzers, respiratory quotient

## Abstract

The use of automated gas exchange analysis systems in exercise studies is common throughout the industrialized world and are frequently used in sports medicine laboratories for the measurement of maximal oxygen uptake (VO_2max_), as an integrative parameter that allows the physical condition to be assessed, in spite of its limitations. Actually, the fundamental principles behind the measurement of respiratory gas exchange (RGE) have not changed for a century. It was used a manual Douglas bag method together with separate chemical analyses. The need for faster and more efficient techniques, has conditioned the traditional procedures and determined the emergence of automated systems. However, the validity and reliability of all these different systems is not well known. The common features associates with these systems, also have disadvantages that must be evaluated at the time of the acquisition of an automated equipment: (1) regular quality control checks, which entails other added economic costs, (2) the validity and reliability of the results, which it is necessary to verify, and (3) the user does not know the equations that determine the values of oxygen consumption and carbon dioxide production. This work aims to clarify the disadvantages of these automated systems. At maximum intensities, the variation of VO_2max_ or VO_2peak_ can be very significant in athletes and even more relevant in sick people undergoing a training program. Therefore, considerable care is needed when comparing RGE data with automated systems.

**New and Noteworthy:**

Actually, stress tests are more conveniently performed with automated systems. It is necessary to examine the validity and reliability of automated respiratory gas exchange systems. The algorithms incorporated in the software, apart from being a “mystery,” show differences with respect to the data provided.

## Introduction

Ergospirometry (EE) is the “coupling” of two methods of functional assessment. On the one hand, “ergometry” (from the Greek root *ergon* = work and the Latin root *metrum* = measurement) constitutes the procedure for measuring mechanical external work. On the other hand, “spirometry” (from the Latin root *spirare* = breathing and *metrum* = measurement) allows the measurement of the volumes and capacities of the respiratory system. In fact, the “spirometric part” should be called “respiratory gas exchange” (RGE) and began to be used to indirectly determine the heat generated in animals and humans (indirect calorimetry). The interested reader can consult review articles on the historical evolution of ergometry (Hollmann and Prinz, 1997) and, possibly, on the first indirect calorimetry device designed by Joseph von Pettenkofer (Jackson, 2011), which lays the foundations on which Nathan Zuntz develops his famous “portable spirometer”(Gunga and Kirsch, 1995).

There are two central RGE parameters for performance and diagnostic objectives: oxygen consumption ($\dot{\text{V}}$O_2_) and carbon dioxide output ($\dot{\text{V}}$CO_2_). Although the fundamental principles of measurement for $\dot{\text{V}}$O_2_ and $\dot{\text{V}}$CO_2_ are the same as two centuries ago, the automated systems have not only brought undoubted advantages but also some disadvantages. As Macfarlane (2001) rightly points out, *“The computer-managed-systems have become so automated that little knowledge of respiratory physiology is required*” (p 842). In addition, the validity and reliability of the results is questionable. The $\dot{\text{V}}$O_2_ validity/reliability studies of the automated RGE systems range from 0% to 15% or 130–268 ml/min, depending on the intensity (Macfarlane, 2001). Validation studies for portable devices show error percentages between 1.1% and 22% (Macfarlane, 2001).

The variation of VO_2max_ or VO_2peak_ can be very significant in athletes and even more relevant in sick people undergoing a training program. Although the variations in peak VO_2_ with training depend on the characteristics of the program (type of exercise, intensity, frequency and duration) and degree of cardiac involvement, the percentages range between 11% and 20% (Cissik and Johnson, 1972; Garcia-Tabar et al., 2015). Thus, even if the measurements taken before and after a training program were carried out with the same device, a misinterpretation could occur.

The scientific basis of $\dot{\text{V}}$O_2_ (ml⋅min^–1^) and $\dot{\text{V}}$CO_2_ (ml⋅min^–1^) measurement are the following elementary equation:

(1) $$\dot{V}\,O_{2}=\left({\dot{V}}_{I}\cdot F_{I}\,O_{2}\right)-\left({\dot{V}}_{E}\cdot F_{E}\,O_{2}\right)$$

(2) $$\dot{V}\,C\,O_{2}=\left({\dot{V}}_{E}\cdot F_{E}\,C\,O_{2}\right)$$

When $\dot{\text{V}}$_I_ and $\dot{\text{V}}$_E_ are equal (respiratory exchange ratio < 1), Eq. 1 can be simplified:

(3) $$\dot{V}\,O_{2}={\dot{V}}_{E}\cdot \left(F_{1}\,O_{2}-F_{E}\,O_{2}\right)$$

However, both the criteria for establishing a maximal test as to evaluate $\dot{\text{V}}$O_2_ in athletes, $\dot{\text{V}}$_I_ and $\dot{\text{V}}$_E_, are different. As a result, Eq. 2 cannot be applied, since $\dot{\text{V}}$_E_ exceeds $\dot{\text{V}}$_I_. Thus, for about a century, applies a correction based on the widely demonstrated fact that in the steady state the quantities of nitrogen inhaled and exhaled may be assumed to be equal. This correction is incorrectly attributed to Haldane and is known as “Haldane’s transformation.” Actually, it should be called the “Gepper and Zunt transformation,” unfortunately written in German (Geppert and Zuntz, 1888) and, consequently, less “visible” to the scientific society. In this work, we will call the transformation of Haldane the transformation of Gepper and Zunt.

The physiological meaning of maximal oxygen uptake is maximal rates of “catchment” of oxygen (respiratory apparatus), “pumping and distribution” of oxygen (cardiovascular system), “physical” transport of oxygen (hemoglobin) and oxygen utilization (mainly in muscle tissue). This integrative perspective allows us to deal with the limiting factors of $\dot{\text{V}}$O_2__max_, although it is thought that under most circumstances the ability of the heart to transport oxygen to and be accommodated by the working muscle (Capelli et al., 2006).

Mathematically, the integrated analysis can be shown by equating Eq. 3 and the Fick equation for the determination of the mean cardiac output. All the elements that are involved in determining the maximum oxygen consumption, they are: maximum ventilation (VE_max_), maximum cardiac output (Q_max_), hemoglobin concentration (Hb), maximum vasodilatation (MV), and maximum mitochondrial activity.

(4) $$Q=\frac{\dot{V}\,O_{2}}{D\,i\,f_{a-v}\,O_{2}};\mathrm{solve}\,\mathrm{for}\,\dot{V}\,O_{2};\dot{V}\,O_{2}=Q\cdot D\,i\,f_{a-v}\,O_{2}$$

Eq. 4 is incomplete because it does not consider the role of the respiratory apparatus. Therefore, it is the aim of this paper review to the drawbacks of automated devices, since there are obvious advantages (ease of calibration and use, considerable information processed into data or graphically).

## General Principles of the Respiratory Gas Exchange Measurement

The objective of this work is not to describe the devices used to determine the ventilation and the composition of exhaled air. The interested reader can consult the excellent book by McLean and Tobin (1987) and calibration studies (Carpenter, 1923; Sparkes, 1968; Nelson, 1971; Yeh et al., 1987; Nelson et al., 1990). The devices actually only measure 5 variables: ventilation ($\dot{\text{V}}$_E_ and $\dot{\text{V}}$_I_), the inspired oxygen fraction (F_I_O_2_), the expired oxygen fraction (F_E_O_2_), the exhaled carbon dioxide fraction (F_E_CO_2_), and the respiratory rate and the heart rate obtained by electrocardiographic recording. Table 1 shows some of the derived parameters that are used more commonly in the evaluation of an ergospirometric test. From these 5 parameters, the automated analyzers provide more than 40 variables, applied in fields such as neumology, cardiology, sports medicine, intensive care, rehabilitation, occupational medicine and nutrition.

**TABLE 1:** Commonly used variables in the evaluation of ergospirometric tests.

**Variable**	**Method of obtaining**
Tidal volume (ml) (V_T_)	Dividing the V_T_ by the respiratory frequency (B_F_)
Carbon dioxide output (ml/min) ($\dot{\text{V}}$CO_2_)	Eq 2
Absolute oxygen consumption (l/min or ml/min) ($\dot{\text{V}}$O_2_ abs)	Eqs 1 or 3
Relative oxygen output (ml/kg/min) ($\dot{\text{V}}$O_2_ rel)	Dividing absolute oxygen consumption by body weight
Respiratory exchange ratio (RER)	Dividing carbon dioxide output by absolute oxygen consumption
Oxygen pulse (ml/heartbeat) (O_2_ pulse)	Dividing the oxygen consumption by the heart rate
Metabolic unit (Met)	Dividing relative oxygen consumption by metabolic unit (3,5 ml/kg/min = 1 Met)
Oxygen respiratory equivalent ($\dot{\text{V}}$_E_/$\dot{\text{V}}$O_2_)	Dividing ventilation by absolute oxygen consumption (ml/min)
Carbon dioxide equivalent ($\dot{\text{V}}$_E_/$\dot{\text{V}}$CO_2_)	Dividing ventilation by carbon dioxide output
Tele-expiratory oxygen pressure (mm Hg) (PET O_2_)	Direct measurement in some devices
Tele-expiratory carbon dioxide pressure (mm Hg)	Direct measurement in some devices
Total respiratory time (seg) (T_T_)	Inverse of respiratory frequency
Inspiratory time (seg) (T_i_)	Direct measurement
Expiratory time (seg) (T_e_)	Direct measurement
Inspiratory central generator (ml/seg) (V_T_/T_i_)	Dividing tidal volume by inspiratory time
Inspiratory switch-off (T_T_/T_i_)	Dividing total respiratory time by inspiratory time

### Gepper and Zunt Transformation

Briefly, the transformation of Gepper and Zunt is analyzed. More complete information can be found in concrete monographs (Consolazi et al., 1963; Otis, 1964) and specific articles (Fox and Bowers, 1973; Wagner et al., 1973; Wilmore and Costill, 1973; Poole and Whipp, 1988) related to the so-called Haldane transformation (Haldane and Graham, 1935). The net rate of exchange of any gas is the difference between the amount inspired and the amount expired per unit of time. Eqs 1 and 2 allow the simple calculation of the rate of exchange of oxygen and carbon dioxide. Since, in steady state, the body neither produces nor consumes nitrogen, the rate of exchange of nitrogen in steady state conditions is zero, so that:

(5) $$F_{1}\,N_{2}\cdot {\dot{V}}_{I}=F_{E}\,N_{2}\cdot {\dot{V}}_{E};{\dot{V}}_{I}=\frac{F_{E}\,N_{2}}{F_{I}\,N_{2}}$$

This simple relationships thought by Geppert and Zunt allows the calculation of oxygen consumption and carbon dioxide production when the volume expired per minute and composition of inspired and expired air are known.

(6) $$\dot{V}\,O_{2}=\left(F_{1}\,O_{2}\cdot \frac{F_{E}\,N_{2}}{F_{1}\,N_{2}}-F_{E}\,O_{2}\right)\cdot {\dot{V}}_{E}$$

(7) $$\dot{V}\,C\,O_{2}=\left(F_{E}\,C\,O_{2}-\frac{F_{E}\,N_{2}}{F_{1}\,N_{2}}\cdot F_{1}\,C\,O_{2}\right)\cdot {\dot{V}}_{E}$$

Since the conditions of measurement of the gas volume involved may vary widely in different circumstances, it is necessary to correct the measured values to standard conditions of temperature and pressure (°C, 760 mm Hg, dry). Thus, while ventilation is measured under body temperature and pressure, saturated conditions (BTPS), $\dot{\text{V}}$O_2_ and $\dot{\text{V}}$CO_2_ are expressed in standard temperature and pressure, dry (STPD). However, this does not constitute any problem in the automated devices, since all have the algorithms to perform the transformations from BTPS to STPD.

### Problems of the Application of Transfer Equations for Respiratory Gas Exchange Analysis

**FIGURE 1:**
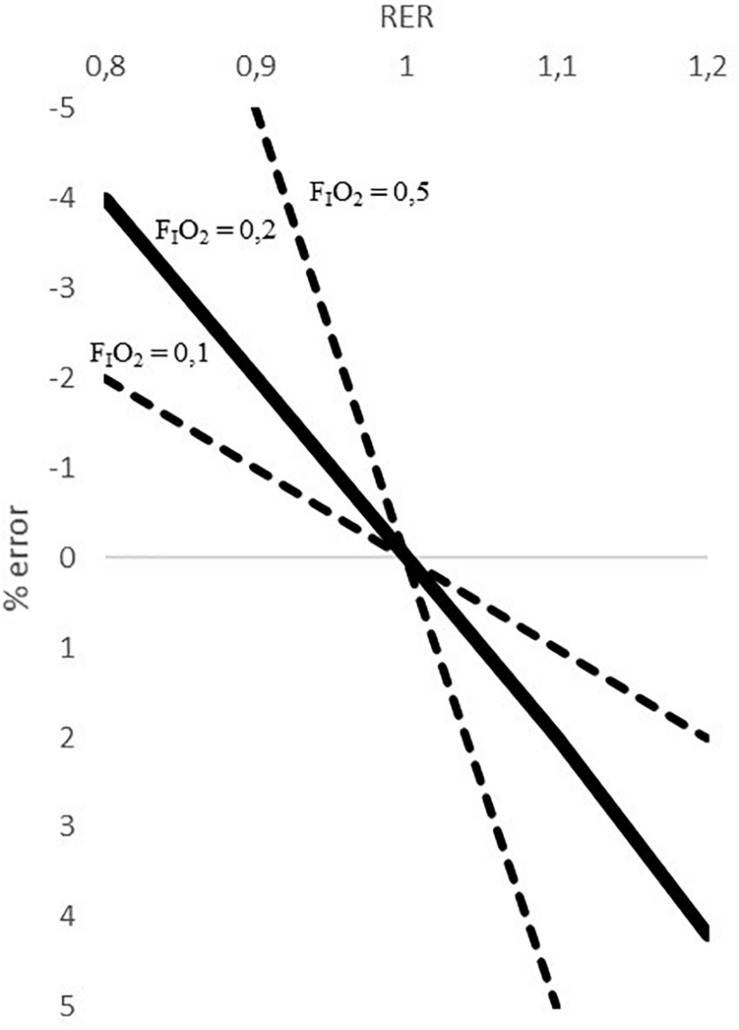
Error involved when oxygen consumption is estimated by Eq. 3 modified from Otis (1964).

When $\dot{\text{V}}$O_2_ is measured by Eq. 3, which ignores the inequality between inspired and expired volumes, the error involved depends on the magnitudes of the respiratory quotient (RQ) and the F_I_O_2_. Figure 1 shows the errors made when using Eq. 3 in relation to the RQ and the F_I_O_2_ (Geppert and Zuntz, 1888). For a RQ equal to 1, Eq. 3 does not show any error regardless of the F_I_O_2_. However, for a fixed value of F_I_O_2_, the error increases as RQ deviates from unity, and for a fixed value of RQ other than unity, the error increases values of F_I_O_2_. For a F_I_O_2_ = 0.2 (close to ambient air; F_I_O_2_ = 20.9) the error cannot be assumed if Eq. 3 is applied. For example, for a RQ of 1.2 a $\dot{\text{V}}$O_2__max_ of 4350 ml/min would imply an error of 4%, which is 4524 ml/min. Error involved when oxygen consumption is estimated by Eq. 3. Modified from Otis (1964), Figure 1.

The other problem has been to consider if it is appropriate or not to apply the transformation of Gepper and Zunt to the determination of pulmonary gas exchange. The controversy is considerable. The studies of Cissik and Johnson (1972) seriously question the use of the Haldane transformation. Concretely they point out *“all experiments evidence available since 1960 indicates that the classic open-circuit method of indirect calorimetry is invalid”* (Haldane and Graham, 1935) (page 757) and “*virtually every measurement of O_2_ consumption by the open-circuit method published in the literature is substantially in error*” (Cissik and Johnson, 1972) (page 590). If these considerations are performed in a resting state (indirect calorimetry), it is more reasonable to think that they are valid in extreme exercise, such as the one carried out in sports medicine and exercise prescription for cardiopulmonary diseases. However, the transformation of Gepper and Zunt has been validated during the exercise performed on a treadmill by Wagner et al. (1973); Wilmore and Costill (1973), although, certainly, the exercise developed by the participants in both studies was not the maximum, considering the criteria of different researchers summarized by Howley et al. (1995). For all the above, some researchers have proposed to make corrections to the transformation of Gepper and Zunt. Because of the ease of processing the data in computer programs, Toien (2013) prefers the mathematical correction of the Gepper and Zunt transformation for measurements on animals that estimate metabolic rate and fuel use, one of the fields of application of the automated devices.

## Possible Disadvantages of Automated Gas Exchange Analysis Systems

From the middle of the last century, a number of electronic devices that incorporate gas analyzers and a variety of flow meters integrated with computers to produce a dedicated respiratory gas exchange (RGE) analysis have been introduced. The greater availability of these commercial apparatuses have, undoubtedly, many advantages over traditional methods of measuring pulmonary gas exchange, such as (1) ease of handling and customization, (2) efficiency in data management for research and clinical diagnostics, and (3) possibility of providing breath-to-breath or averaged data at the user’s interest. However, the disadvantages must also be considered. In my own experience, one of the most important disadvantages of automated systems is the manufacturers and companies that market these very expensive devices are not transparent with regards to the functioning of the hardware and, above all, the software. As Macfarlane (2001) rightly points out, “*Many fully automated system have become a “black box” which can generate high densities of data without the user having sufficient understanding*…*”* (p 851).

If we take into account Eqs 2 and 3, we can take into consideration the problems of automated devices:

(1) First, problems can be caused by the measurement systems of ventilation and gas fractions, both in inspired and exhaled air. Virtually all companies that market automated gas analysis equipment ensure the validity and reliability of measuring equipment. Nevertheless, despite certifying the linearity of ventilation measuring devices, it is not certain that it will remain in a wide range, especially in athletes who can mobilize around 60% to 70% of their forced vital capacity. The question of aligning the flow meters is fundamental, as errors in the values of maximum oxygen consumption or peak oxygen consumption can occur. The possible errors in the oxygen and carbon dioxide analyzers are not in our opinion so determinant.
(2) Second, as Macfarland points out, to the fact that the different software of the various undertakings marketing those automated devices may be regarded as black boxes. Certainly, at present, many companies in the sector seem to have been “unified” or “grouped.” It would seem that the companies have unified the software. We have our doubts that this has been the case.

The second question is then dealt with in a simple way, essentially. As mentioned above, the validity and reliability of the devices are dealt with in books (McLean and Tobin, 1987) and specific articles (Sparkes, 1968; Porszasz et al., 1994; Macfarlane, 2001) on the subject.

### Validity and Reliability Measuring Devices (Flow Meters and Gas Analyzers)

Although, in reality, to measure volume of gases rather than gas flow, the different devices, mainly pneumotachographs and turbines, create measurement problems at high current volumes and respiratory frequency during maximal exercise. In addition, possible errors can be caused by leakage and frictional resistances. Turbine system controls to measure ventilation have proven extremely accurate over a wide range of flows, both continuous and discontinuous. The pneumotachographs, originating from the Fleisch apparatus, have undergone many design variations and corrections by means of software, to avoid the alignment and the inherent problems (pressure, temperature, and humidity) of the laws of gases.

On the other hand, the monitoring of gases on a single apparatus (mass spectometer) has very high economic and maintenance costs, so their usage (many laboratories) is prohibitive. Thus, modern devices separately measure the essential ones to determine oxygen consumption and carbon dioxide production (see Eqs 2 and 3). The measurement of F_E_CO_2_ is accurate, although it is necessary to watch out for possible contamination of the sampling chamber. Different systems are used for it (electrochemical, paramagnetic, and Zirconium cell) that, according to different studies, are very accurate and therefore valid and reliable.

### The “Mystery” of the Equations for Determining $\dot{\text{V}}$O_2_ and $\dot{\text{V}}$Co_2_

For the last 10 years, we have been trying to get Jaeger, through its distributor in Spain, to provide us with the equations used by the software to determine $\dot{\text{V}}$O_2_ and $\dot{\text{V}}$CO_2_. We thought that the system could not use different equations until the values of the RQ = 1 and another algorithm when RQ > 1. We think that many of these software systems use the following equations:

(8) $$\dot{V}\,O_{2}=\left({\dot{V}}_{E}\cdot K\,H\,\frac{F_{I}\,O_{2}}{100}-{\dot{V}}_{E}\cdot \frac{F_{E}\,O_{2}}{100}\right)\cdot K\,B\,S$$

(9) $$\dot{V}\,\text{C}\,O_{2}=\left({\dot{V}}_{E}\cdot \frac{F_{E}\,C\,O_{2}}{100}-{\dot{V}}_{E}\cdot K\,H\cdot \frac{F_{I}\,C\,O_{2}}{100}\right)\cdot K\,B\,S$$

Where KH is a transformation of the equation of Gepper and Zunt (Haldane transformation) *KH* = (100−*F*_*E*_*O*_2_−*F*_*E*_*CO*_2_)/(100−*F*_*I*_*O*_2_−*F*_*I*_*CO*_2_) and KBS is the correction factor for converting BTPS to STPD conditions $K\,B\,S=\frac{A\,P\,i\,n\,m\,m\,H\,g-47}{863}$; *AT* = *atmosphericpressure*

By entering data into the equations provided by the software, there are differences from the “real” data values. The important question is not that there are differences between the values provided by the device and those calculated by Eqs 7 and 8, but that the error fluctuates between −1 and 27 for $\dot{\text{V}}$O_2_ (ml⋅Kg^–1^⋅min^–1^) and 0 and 23 for $\dot{\text{V}}$CO_2_ (ml⋅Kg^–1^⋅min^–1^). We understand that the companies that commercialize the different automated devices do not want their algorithms and adjustments to become popular, so that the competition could not make use of it. A possible explanation is that the data are provided in a certain time or breath-by-breath. The sampling interval used to report the data are important. The variability has been shown to be higher when the shorter sampling interval is used. It is suggested that an average of 15–20 s be used, as they produce similar variabilities but allow a high degree of precision (Robergs et al., 2010). Firstly, it is not logical to think that physiological changes occur in “one breath” or even “one set of breaths.” The physiological variations that occur during exercise are a process, not a specific moment. Second, the breath-by-breath systems present the problem of “synchronizing” the ventilation measurement and gas analysis, so that the delay in time between both can cause up to 30% error in $\dot{\text{V}}$O_2__max_ at high breath frequencies (Sainsbury et al., 1988; Hughson et al., 1991; Proctor and Beck, 1996).

Can these mistakes be assumed? In our opinion, they are not admissible when an endurance athlete is evaluated. One of the central parameters of these athletes is the VO_2__max_. Certainly, the intra-individual day-to-day variations oscillate between 1% and 12% in the same lab and up to a 15% difference when testing in different laboratories (Gunga and Kirsch, 1995). In our opinion, intra-individual differences are related to the fact that maximum oxygen consumption is a parameter that integrates various functions (VE_max_, Q_max_, Hb, MV, and MMA).

In short, automated devices have undoubted advantages, but when high precision is required, such as in sports medicine or research centers, the source of errors can be significant. After almost 100 years of technological development, in some laboratories the air is still collected in Douglas sacks and the air composition is analyzed by means of the Haladane or Scholander apparatus. These methods are very laborious but more exact (higher validity and reliability) and economical, although certainly not free of problems. Nevertheless, there are more variations between automated systems than traditional methods and, therefore, the possibility of greater variability in measurement of $\dot{\text{V}}$O_2__max_ when using the automated systems compared with the traditional methods. Therefore, considerable care is needed when comparing RGE data with automated systems. Regardless of the extreme care in calibration that must be taken, automated devices have two main disadvantages:

(1) Mainly the alignment of flow meters and also of the analyzers. It is true that alignment can be corrected by algorithms. But at maximum exercise intensities, these corrections may not be valid.
(2) The “ultrasecret” that different companies have to make known the algorithms with which they calculate the $\dot{\text{V}}$O_2__max_ and the $\dot{\text{V}}$CO_2__max_. In our opinion, knowing the algorithms would allow the user to detect possible errors in the data provided by the automated devices, especially from the point of view of the teaching of gas exchange during the exercise. Small errors in measurement of FEO2 and VE can cause differences between 4% and 23% in the VO_2_ values calculated by computerized devices with respect to those calculated by Eqs 3 and 6.

## Data Availability Statement

All datasets analyzed for this study are included in the manuscript.

## Author Contributions

FC-M contributed to the conception or design of the work. JR-Á, IL-C, and FC-M contributed to the acquisition, analysis, or interpretation of data for the work, drafted the manuscript, and critical review of the manuscript. All authors gave final approval and agreed to be accountable for all aspects of work ensuring integrity and accuracy.

## Conflict of Interest

The authors declare that the research was conducted in the absence of any commercial or financial relationships that could be construed as a potential conflict of interest.

## References

[B1] Capelli C. Antonutto G. Kenfack M. A. Cautero M. Lador F. Moia C. (2006). Factors determining the time course of VO2(max) decay during bedrest: implications for VO2(max) limitation. *Eur. J. Appl. Physiol.* 98 152–160. 10.1007/s00421-006-0252-3 16924528

[B2] Carpenter T. M. (1923). An apparatus for the exact analysis of air in metabolism investigations with respiratory exchange chambers. *J. Metab. Res.* 4 1–25.

[B3] Cissik J. H. Johnson R. E. (1972). Myth of nitrogen equality in respiration: its history and implications. *Aerosp. Med.* 43 755–758. 4560164

[B4] Consolazi C. F. Johnson R. E. Pecora L. J. (1963). *Physiological Measurement of Metabolic Functions in Man.* New York, NY: McGraw Hill.

[B5] Fox E. L. Bowers R. W. (1973). Steady-state equality of respiratory gaseous N 2 in resting man. *J. Appl. Physiol.* 35 143–144. 10.1152/jappl.1973.35.1.143 4716149

[B6] Garcia-Tabar I. Eclache J. P. Aramendi J. F. Gorostiaga E. M. (2015). Gas analyzer’s drift leads to systematic error in maximal oxygen uptake and maximal respiratory exchange ratio determination. *Front. Physiol.* 6:308. 10.3389/fphys.2015.00308 26578980 PMC4626835

[B7] Geppert J. Zuntz N. (1888). Ueber die regulation der atmung. *Arch. Gesamte Physiol. Menschen Tiere* 42 189–245.

[B8] Gunga H. C. Kirsch K. A. (1995). Nathan Zuntz (1847-1920)–a German pioneer in high altitude physiology and aviation medicine. Part II: Scientific work. *Aviat. Space Environ. Med.* 66 172–176. 7726784

[B9] Haldane J. S. Graham J. I. (1935). *Methods of Air Analysis.* Lincoln: Anybook Ltd.

[B10] Hollmann W. Prinz J. P. (1997). Ergospirometry and its history. *Sports Med.* 23 93–105. 10.2165/00007256-199723020-00003 9068094

[B11] Howley E. T. Bassett D. R. Jr. Welch H. G. (1995). Criteria for maximal oxygen uptake: review and commentary. *Med. Sci. Sports Exerc.* 27 1292–1301. 8531628

[B12] Hughson R. L. Northey D. R. Xing H. C. Dietrich B. H. Cochrane J. E. (1991). Alignment of ventilation and gas fraction for breath-by-breath respiratory gas exchange calculations in exercise. *Comput. Biomed. Res.* 24 118–128. 10.1016/0010-4809(91)90024-q 2036778

[B13] Jackson D. C. (2011). Academic genealogy and direct calorimetry: a personal account. *Adv. Physiol. Educ.* 35 120–127. 10.1152/advan.00121.2010 21652494

[B14] Macfarlane D. J. (2001). Automated metabolic gas analysis systems: a review. *Sports Med.* 31 841–861. 10.2165/00007256-200131120-00002 11665912

[B15] McLean J. A. Tobin G. (1987). *Animal and Human Calorimetry.* Cambridge: Cambridge University Press.

[B16] Nelson G. O. (1971). *Controlled Test Atmospheres: Principles and Techniques.* Ann Arbor, MI: Ann Arbor Science Publishers.

[B17] Nelson S. B. Gardner R. M. Crapo R. O. Jensen R. L. (1990). Performance evaluation of contemporary spirometers. *Chest* 97 288–297. 10.1378/chest.97.2.288 2298052

[B18] Otis A. B. (1964). “Quantitative relationships in steady-state gas exchange,” in *Handbook of Physiology*, eds Fin O. W. Rahn H. (Washington, DC: The American Psychological Society), 681–698.

[B19] Poole D. C. Whipp B. J. (1988). Haldane transformation. *Med. Sci. Sports Exerc.* 20 420–421. 3173054

[B20] Porszasz J. Barstow T. J. Wasserman K. (1994). Evaluation of a symmetrically disposed Pitot tube flowmeter for measuring gas flow during exercise. *J. Appl. Physiol.* 77 2659–2665. 10.1152/jappl.1994.77.6.2659 7896605

[B21] Proctor D. N. Beck K. C. (1996). Delay time adjustments to minimize errors in breath-by-breath measurement of Vo2 during exercise. *J. Appl. Physiol.* 81 2495–2499. 10.1152/jappl.1996.81.6.2495 9018497

[B22] Robergs R. A. Dwyer D. Astorino T. (2010). Recommendations for improved data processing from expired gas analysis indirect calorimetry. *Sports Med.* 40 95–111. 10.2165/11319670-000000000-00000 20092364

[B23] Sainsbury D. A. Gore C. J. Withers R. T. Ilsley A. H. (1988). An on-line microcomputer program for the monitoring of physiological variables during rest and exercise. *Comput. Biol. Med.* 18 17–24. 10.1016/0010-4825(88)90052-2 3335124

[B24] Sparkes D. W. (1968). A standard choked nozzle for absolute calibration of air flowmeters. *Aeronaut. J.* 72 335–338. 10.1017/s0001924000084141

[B25] Toien O. (2013). Automated open flow respirometry in continuous and long-term measurements: design and principles. *J. Appl. Physiol.* 114 1094–1107. 10.1152/japplphysiol.01494.2012 23349455

[B26] Wagner J. A. Horvath S. M. Dahms T. E. Reed S. (1973). Validation of open-circuit method for the determination of oxygen consumption. *J. Appl. Physiol.* 34 859–863. 10.1152/jappl.1973.34.6.859 4711599

[B27] Wilmore J. H. Costill D. L. (1973). Adequacy of the Haldane transformation in the computation of exercise V O2 in man. *J. Appl. Physiol.* 35 85–89. 10.1152/jappl.1973.35.1.85 4716166

[B28] Yeh M. P. Adams T. D. Gardner R. M. Yanowitz F. G. (1987). Turbine flowmeter vs. Fleisch pneumotachometer: a comparative study for exercise testing. *J. Appl. Physiol.* 63 1289–1295. 10.1152/jappl.1987.63.3.1289 3115953

